# Hospital discharge documentation of a designated clinician for follow-up care and 30-day outcomes in hip fracture and stroke patients discharged to sub-acute care

**DOI:** 10.1186/s12913-018-2907-2

**Published:** 2018-02-09

**Authors:** Andrea L. Gilmore-Bykovskyi, Korey A. Kennelty, Eva DuGoff, Amy J. H. Kind

**Affiliations:** 1 University of Wisconsin-Madison School of Nursing, 701 Highland Ave, Madison, WI 53705 USA; 2 Department of Medicine, Division of Geriatrics, University of Wisconsin-Madison School of Medicine & Public Health, 750 Highland Ave, Madison, WI 53726 USA; 3 William S. Middleton Memorial Veterans Hospital Geriatric Research Education and Clinical Center (GRECC), 2500 Overlook Terrace, Madison, WI 53705 USA; 4 University of Iowa College of Pharmacy, Division of Health Services Research, 115 S. Grand Ave, Iowa City, IA 52242 USA; 5 University of Wisconsin-Madison School of Pharmacy, 777 Highland Ave, Madison, WI 53705 USA; 6 Department of Population Health Sciences, University of Wisconsin-Madison School of Medicine & Public Health, 610 N Walnut St., 707 WARF Building, Madison, WI 53726 USA; 7 UW-Madison School of Nursing, 3173 Cooper Hall, 701 Highland Avenue, Madison, WI 53705 USA

**Keywords:** Discharge communication, Follow-up care, Rehospitalization, Transitional care

## Abstract

**Background:**

Transitions to sub-acute care are regularly complicated by inadequate discharge communication, which is exacerbated by a lack of clarity regarding accountability for important follow-up care. Patients discharged to sub-acute care often have complex medical conditions and are at heightened risk for poor post-hospital outcomes, yet many do not see a provider until 30 days post discharge due to current standards in Medicare regulations. Lack of designation of a responsible clinician or clinic for follow-up care may adversely impact patient outcomes, but the magnitude of this potential impact has not been previously studied.

**Methods:**

We examined the association of designating a responsible clinician/clinic for post-hospital follow-up care within the hospital discharge summary on risk for 30-day rehospitalization and/or death in stroke and hip fracture patients discharged to sub-acute care. This retrospective cohort study used Medicare Claims and Electronic Health Record data to identify non-hospice Medicare beneficiaries with primary discharge diagnoses of stroke/ or hip fracture discharged from one of two urban hospitals to sub-acute care facilities during 2003–2008 (*N* = 1130). We evaluated the association of omission of the designation of a responsible clinician/clinic for follow-up care in the hospital discharge summary on the composite outcome of 30-day rehospitalization and/or death after adjusting for patient characteristics and utilization. We used multivariate logistic regression robust estimates clustered by discharging hospital.

**Results:**

Patients whose discharge summaries omitted designation of a responsible clinician/clinic for follow-up care were significantly more likely to experience 30-day rehospitalization and/or death (OR: 1.51, 95% CI 1.07–2.12, *P* = 0.014).

**Conclusions:**

The current study found a strong relationship between the omission of a responsible clinician/clinic for follow-up care from the hospital discharge summary and the poor outcomes for patients transferred to sub-acute care. More research is needed to understand the role and impact of designating accountability for follow-up care needs on patient outcomes.

## Background

Research suggests that patients who are discharged from hospitals to sub-acute care facilities are at increased risk for experiencing 30-day rehospitalization and death [1–3]. This is widely considered to reflect deficits in care coordination that may be exacerbated by inadequate or inaccurate communication between care settings [4–6], and can lead to significant distress for patients and their caregivers [7, 8]. In addition to being costly, it is believed that many of these significant negative outcomes, which disproportionality affect sub-acute care populations, may be avoidable [9, 10]. As such, both hospitals and sub-acute care facilities are facing increasing pressure to improve care for this population in the way of existing monetary penalties incurred by hospitals for high 30-day re-hospitalization and new quality measures imposed on skilled nursing facilities for both 30-day emergency room visits and rehospitalizations [11].

These adverse outcomes may be driven in part by inadequate discharge communication between the hospital and sub-acute care facility, particularly regarding which provider is accountable for follow-up care. Both hospital and sub-acute care providers have identified lack of clarity regarding accountability for important follow-up care needs such as pending laboratory tests and lack of well-established, bidirectional communication routes between settings as common barriers to effective care coordination [12, 13]. Uncertainty regarding which clinician or clinic is responsible for providing follow-up care and clarifying medical orders may contribute to care delays and poor patient outcomes in the initial post-discharge period [12]. Clearly identifying which provider or clinic is responsible for follow-up care is particularly important in facilitating continuity of the medical plan of care. Prior studies suggest that improved discharge communication and medical follow-up are important factors in reducing 30-day rehospitalization in general medical and heart failure-specific populations discharged to community settings, but sub-acute care populations remain under-studied [14, 15].

Accountability may be even more important to sub-acute care populations who are more likely to experience cognitive vulnerabilities and medical complexity [16]. While experts agree that establishing clear accountability for follow-up in care is important for high quality care transitions [17], the potential impact of designation of a responsible clinician for follow-up care on sub-acute populations has not been examined. Although many sub-acute care patients have complex chronic and acute conditions which may require care from various specialty providers, and nearly half have cognitive impairment [16], they may not be seen by a medical provider for up to 30 days post discharge due to minimum standards in current Medicare regulations even when receiving skilled nursing care [18]. This standard varies by state with some requiring an initial visit within 48–72 h [19]. However, in many skilled nursing facility (SNF) settings, there is not always an on-site physician or advanced practice provider to navigate urgent issues that occur in the early post-discharge period. Additionally, requirements for initial admission visits commonly focus on signing admitting orders and completing a general medical evaluation, as such, these visits do not necessarily serve as a replacement for other requisite medical follow-up appointments such as post-surgical visits [18]. Consequentially, the hospital discharge summary plays a critical role in clarifying and facilitating proper follow-up care for these patients, as it is the primary communication tool and also serves as the document from which sub-acute care admission orders are transcribed [12].

The objective of this study was to determine the potential impact of omission of designation of a responsible clinician/clinic in hospital discharge summary on risk for 30-day rehospitalization and/or death in a population of patients discharged to sub-acute care.

## Methods

### Study sample and Medicare data source/linkage

This retrospective cohort study included all Medicare beneficiaries 18 years and older discharged to sub-acute care under the Medicare skilled nursing benefit with primary discharge diagnoses of hip fracture or stroke from one of two Midwestern hospitals between 2003 and 2009. This level of care is commonly referred to as “skilled nursing care” in the United States, and is typically provided within skilled nursing facilities (which are commonly housed within a nursing home facility) and inpatient rehabilitation facilities. To qualify for skilled nursing care, Medicare beneficiaries must have experienced a qualifying hospital stay of at least 3-days inpatient and had a provider certify that they require daily skilled care due to a hospital-related condition. Discharge disposition was identified using administrative data that are mandatorily compiled by hospital case managers and was further verified through presence of a SNF claim on the day of discharge or day following discharge. Diagnoses were identified using International Classification of Diseases, 9th edition (ICD-9) diagnosis codes of 431, 432, 434, and 436 for stroke; and 805.6, 805.7, 806.6, 806.7, 808, and 820 for hip fractures. Hospital administrative data compiled for all study patients prior to discharge were used to identify discharges to sub-acute care facilities. Medicare claims data for each beneficiary were obtained from the Medicare Chronic Condition Warehouse.

Prior to exclusions, the initial sample size was 1223. Twelve patients were excluded because they did not have a discharge summary; 17 because they were not discharged to sub-acute care upon record review; 49 had a primary diagnosis other than stroke/hip fracture; and 15 were discharged to hospice or comfort care, for a final sample of 1130 patients. The University of Wisconsin-Madison Institutional Review Board approved the study and waived the requirement for informed consent.

### Variables

The primary explanatory variable was the presence or absence of a responsible clinician/clinic designated for medical follow-up*,* and was defined as discharge summary documentation of a specific professional’s name (e.g. “Dr. Jones”), professional type (e.g. primary care provider, neurologist), or clinic (e.g. neurology clinic) for medical follow up within any timeframe during the post-hospital period. The definition was purposefully broad to ensure that all attempts to designate a clinician/clinic for follow-up within the discharge summary were captured. In the jurisdiction in which this study was conducted, the hospital discharge summary documents were dictated by a clinician into a telephone–based dictation system, transcribed, and sent electronically to the attending physician/ provider for review and signature. The format for discharge summary creation was unstructured and did not include mandatory or standardized fields for the provider to address. For the present sample, 85% of all discharge summaries were completed within 24 h following discharge and the mean date of creation for those completed after 24 h following discharge was 5 days (range 2 to 15). The primary explanatory variable was abstracted from each patient’s discharge summary by two trained medical abstractors utilizing a standardized abstraction protocol, manual, and form [20]. Abstractors independently reviewed different cases and a 10% random re-abstraction for this variable yielded 96% inter-rater agreement.

All other variables were created using Medicare claims data. The outcome variable was a composite variable defined as any rehospitalization within 30 days of discharge from the patient’s index hospitalization and/or death within 30 days of discharge. The outcome variables were combined for both power and theoretical reasons, as both are markers of 30-day post-hospital care quality. Rehospitalization was defined based on Medicare’s definition of 30 day rehospitalization, and represented any acute care stay that was not within a hospital specialty unit (i.e. psychiatric unit or cancer hospital), inpatient rehabilitation hospital, or long-term care hospital and not for rehabilitation (DRG 462) [1]. Dates of death were collected using the Medicare denominator file.

Control variables included patient sociodemographics including age at index hospitalization, sex, and Medicaid enrollment status. Non-Caucasian race was grouped into one combined category due to the predominance of Caucasians within the study sample (97%) reflecting regional hospital population characteristics. The index hospital length of stay variable was highly skewed and consequentially was divided into categories (i.e., 1–4, 5–6, 7–9, > 10 days). Disease severity during the index hospitalization was represented using an indicator variable for mechanical ventilation (CPT 94656, 94,657; ICD-9 96.7×) [21] and/or placement or revision of a gastrostomy tube during the index hospitalization (CPT 43750, 43,760,

43,761, 43,832, 43,246; ICD-9 43.11). We use the Elixhauser Comorbidity Score, which is a widely-used comorbidity index based on the Elixhauser classification system to capture an individual’s chronic disease burden [22]. Further, the Centers for Medicare and Medicaid Services (CMS) new enrollee hierarchical condition category (HCC) score was included as a measure of utilization risk adjustment [23].

### Analysis

We first examined the bivariate differences in the patient characteristics by designation of a responsible clinician/clinic on the discharge summary using chi-square tests for categorical variables and t-tests for continuous variables after evaluating variables for normal distributions. We used a multivariate logistic regression model with robust estimates to estimate the association between omitted designation of a responsible clinician/clinic for follow-up within the hospital discharge summary and occurrence of the combined outcome of 30-day rehospitalization and/or death. Analysis was clustered by the discharging hospital. Patients who visit the same hospital may be similar to each other due to area of residence or disease characteristics, so we accounted for this clustering using robust standard errors. We adjusted for patient characteristics including patient age at index hospitalization, gender, Medicaid status, CMS-HCC score, index hospital length of stay, presence of mechanical ventilation or gastrostomy tube placement during index hospitalization, and Elixhauser comorbidity score. A 2-sided *P* < .05 was used to establish statistical significance and report odds ratios for the primary outcome variable with 95% confidence intervals (CI). Statistical analyses were conducted using SAS version 9.2 and Stata version 13.1.

## Results

Overall, the average age at index hospitalization was 80.8 years, with average index hospital lengths of stay of 6.5 days (Table 1). The majority (83%) of patients had discharge summaries designating a responsible clinician/clinic for follow-up. Most patient characteristics demonstrated little difference between patients with and without designation of a responsible clinician/clinic for medical follow-up within their discharge summary. Seventeen percent of the sample experienced the combined outcome of 30-day rehospitalization and/or death.

**Table 1:** Key sample characteristics^a^ (*N* = 1130)

Characteristic	Overall (*N* = 1130)	Discharge communication elements present specifying clinician/clinic for follow-up in hospital discharge summary		
		Designation present (*n* = 932)	Designation omitted (*n* = 198)	*P*-value
*Patient sociodemographic characteristics*				
Average age in years at discharge (standard deviation)	80.84 (9.37)	80.64 (9.64)	81.77 (7.97)	0.122
< 65 years	53	49 (5.26)	4 (2.02)	0.28
65–69 years	75	66 (7.08)	9 (4.55)	
70–74 years	112	90 (9.66)	22 (11.11)	
75–79 years	184	153 (16.42)	31 (15.66)	
80–84 years	260	211 (22.64)	49 (24.75)	
≥ 85 years	446	363 (38.95)	83 (41.92)	
Female	765	640 (68.67)	125 (63.13)	0.130
Medicaid	119	100 (10.73)	19 (9.60)	0.637
Race non-caucasian	26	21 (2.25)	5 (2.53)	0.817
*Patient prior medical history*				
HCC^b^ Score (standard deviation)	1.19 (.25)	1.09 (0.3)	1.06 (0.2)	0.89
Index hospital length of stay in days (standard deviation)	6.53 (4.34)	6.65 (4.5)	5.94 (3.7)	0.04
1–4 days	366	291 (25.75)	75 (6.63)	0.155
5–6 days	238	193 (17.07)	43 (3.8)	
7–9 days	263	225 (19.91)	38 (3.36)	
> 10 days	263	223 (19.73)	40 (3.53)	
Primary diagnosis:				
Stroke	394	284 (30.47)	110 (55.56)	0.000
Hip fracture	736	648 (69.53)	88 (44.44)	0.000
Mechanical ventilation or gastrostomy tube placement during index hospitalization	37	34 (3.65)	3 (1.52)	0.126
Elixhauser comorbidity score (standard deviation)	5.6 (7)	5.6 (7.03)	5.9 (7.04)	0.792
*Outcomes*				
Re-hospitalization within 30 days	148	119 (12.77)	29 (14.65)	0.477
Death within 30 days	60	47 (5.04)	13 (6.57)	0.385
30 Day re-hospitalization and/or death (combined outcome)	198	147 (15.77)	46 (20.71)	0.090

^a^*HCC* New Enrollee Hierarchical Condition Category Score created using the Centers for Medicare and Medicaid Services Methodology

^b^Elixhauser Score = Validated modification of the Elixhauser Comorbidity Measure [10]

After multivariate adjustment, patients with no clinician/clinic designated for follow-up in their discharge summary were more likely to experience 30-day rehospitalization and/or death relative to patients who had a clinician/clinic designated in their hospital discharge summary (20.7% vs. 15.7%; adjusted OR 1.51; 95% CI, 1.07–2.12) (Table 2). The majority of the 30-day outcome events were rehospitalizations (*n* = 148; 74% of outcome events).

**Table 2:** Omission of designating a clinician/clinic for follow-up in discharge summary and odds for 30-day rehospitalization/death (*N* = 1130)

	Adjusted odds ratio^a^ (95% CI)	*P* value
30-day follow-up designation omitted	1.51 (1.07–2.12)	0.014*
Age, mean (SD)	0.97 (0.93–1.0)	0.176
Female	1.18 (0.82–1.68)	0.362
Race, non-white	1.35 (1.09–1.68)	0.006**
Medicaid enrolled	0.51 (0.16–1.57)	0.240
Composite Elixhauser score	1.02 (0.97–1.08)	0.374
Hierarchical condition category score prior to index hospitalization, mean (SD)	5.96 (3.07–11.6)	< 0.001***
Mechanical ventilation or G-tube, index hospitalization	0.9 (0.42–1.97)	0.798
Hospital index length of stay		
1–4 d	Ref	Ref
5–6 d	1.16 (0.69–1.98)	0.569
7–9 d	1.3 (1.13–1.5)	< 0.001***
> 10 d	2.06 (1.3–3.26)	0.002**

^a^Adjusted for all variables listed, clustered at hospital level

*Significant at *p* ≤ 0.05

**Significant at *p* ≤ 0.01

***Significant at *p* ≤ 0.001

## Discussion

Nearly 20% of stroke and hip fracture patients discharged to post-acute care facilities did not have a responsible clinician/clinic for follow-up listed in the hospital discharge summary, and this omission was associated with 60% greater likelihood of 30-day readmission and/or death. This study provides preliminary evidence that documentation clarifying responsibility for post-hospital follow-up care may influence clinical and utilization outcomes for sub-acute care populations. To our knowledge, this is the first study to offer evidence of this possible linkage. The potential role of perceived accountability or the lack of accountability for specific activities in the early post-hospital period merit further examination as a potential mechanism contributing to negative 30-day outcomes. While improved accountability during transitions has been identified as an important element for ensuring safe transitions [17], additional research is needed to explore the role and impact of designating responsibility for specific care activities post-discharge on accountability and clinical outcomes in more diverse settings.

Our findings suggest that future studies should focus on the role of chronic conditions and utilization risk provided the significantly higher adjusted odds of re-admission and/or death based on HCC scores. Past research has found the HCC to be superior in predicting both mortality and hospital admissions [24, 25]. Our findings further suggest that racial/ethnic minorities may also experience less protective benefits from medical follow-up, which suggests that other factors also drive hospital readmission such as community resources, family support and disparities in care quality. Provided the very small sample of non-Caucasians in the present study, it was not feasible to further examine the relationships between these characteristics and 30-day outcomes. However, future well-powered studies are needed to examine the contributive role of utilization risk, multiple chronic conditions and race/ethnicity in modifying the potentially protective role of post-discharge medical follow-up.

Research on documentation within the discharge summary has consistently found that they are largely non-standardized documents with variable quality and completeness [6, 26]. This is perhaps due in part to a lack of regulatory mandates regarding discharge summary content. None of the major regulatory bodies, including The Joint Commission (TJC) and CMS, mandate that medical follow-up instructions be included within hospital discharge documentation. TJC mandates that a hospital discharge summary be produced for every patient within 30 days of discharge and requires minimal, vaguely defined standards for the content of these documents. Various expert recommendations for the content of hospital discharge summaries have been published, which all highlight inclusion of follow-up instructions, including identifying a responsible provider/clinic for medical follow-up, as a requisite component for discharge summaries [6, 17, 27, 28]. Existing regulations emphasize a focus on the hospital stay instead of on the post-hospital plan. These findings provide further support for calls for increased attention to the content and completeness of documentation in discharge summaries [17]. Strategies are needed to better support clinicians authoring discharge summaries including important post-hospital care elements such as responsibility for follow-up care within discharge instructions through the development of standardized templates and documentation tools.

While an exploratory study, this study has several noteworthy strengths. We link together primary data on the contents of hospital discharge instructions to secondary administrative data to create gold-standard metrics for rehospitalization and death. There is substantial variation in the content of these hospital discharge summaries, and few studies have linked specific elements with the hospital discharge summary to claims-derived patient outcomes. To our knowledge, this is the first study to do so. Furthermore, we focus on an understudied high-risk group of older adults, namely those discharged to sub-acute care settings for whom high-quality discharge documentation is critical [5].

Interpretation of these findings should be considered in light of a number of study limitations. The sample is drawn from geographically similar area in the Midwest that may not reflect other geographic areas. The use of Medicare data provides a strong record of billed medical events (e.g., rehospitalizations and death), but is limited in its ability to provide in-depth data on disease severity, clinical practice, and patient social factors. We worked to address this data limitation through use of control variables in our models, nevertheless, some unrecognized confounding may remain. The analytic strategies applied in the present study were also limited by statistical power and consequentially this study did not examine multinomial 30-day outcomes or subgroup analysis to better understand whether the relationship between designation of follow-up and 30-day outcomes. Both of these approaches are merited in future well-powered studies. Furthermore, as the goal of this study was to understand real-world implications of omission of designation of follow-up on patient outcomes, we do not match or balance groups based upon observed characteristics and, as such, a causal inference cannot be inferred from the present findings [29]. We used all-cause rehospitalizations as an outcome, however, it is important to note that not all re-hospitalizations can or should be prevented [30].

## Conclusion

These findings suggest that not designating a responsible clinician/clinic for post-hospital follow-up within the hospital discharge summary may be associated with poorer 30-day outcomes in patients discharged to sub-acute care facilities. Further research examining the role and impact of clarifying accountability during the post-discharge period is merited.

## Abbreviations

CI: Confidence Intervals
CMS: Centers for Medicare and Medicaid Services
HCC: Hierarchical Condition Category
ICD-9: International Classification of Diseases, 9th Edition
SNF: Skilled Nursing Facility
TJC: The Joint Commission
